# Leadership Development of Rehabilitation Professionals in a Low-Resource Country: A Transformational Leadership, Project-Based Model

**DOI:** 10.3389/fpubh.2017.00143

**Published:** 2017-06-23

**Authors:** Maureen Romanow Pascal, Monika Mann, Kim Dunleavy, Julia Chevan, Liliane Kirenga, Assuman Nuhu

**Affiliations:** ^1^Department of Physical Therapy, Misericordia University, Dallas, PA, United States; ^2^Department of International Health, Johns Hopkins Bloomberg School of Public Health, Baltimore, MD, United States; ^3^Department of Physical Therapy, University of Florida, Gainesville, FL, United States; ^4^Department of Physical Therapy, Springfield College, Springfield, MA, United States; ^5^Department of Physical Therapy, Rwanda Military Hospital, Kigali, Rwanda; ^6^Department of Physiotherapy, University of Rwanda College of Medicine and Health Sciences, Kigali, Rwanda

**Keywords:** physiotherapy, leadership development, professional development, rehabilitation, transformational leadership, constructivist approach, active learning, Rwanda

## Abstract

**Background and rationale:**

This paper presents an overview of the activities and outcomes of the Leadership Institute (LI), a short-term leadership development professional development course offered to physiotherapists in a low-resource country. Previous studies have provided examples of the benefits of such programs in medicine and nursing, but this has yet to be documented in the rehabilitation literature. The prototype of leadership development presented may provide guidance for similar trainings in other low-resource countries and offer the rehabilitation community an opportunity to build on the model to construct a research agenda around rehabilitation leadership development.

**Pedagogy:**

The course used a constructivist approach to integrate participants’ experiences, background, beliefs, and prior knowledge into the content. Transformational leadership development theory was emphasized with the generation of active learning projects, a key component of the training.

**Outcomes:**

Positive changes after the course included an increase in the number of community outreach activities completed by participants and increased involvement with their professional organization. Thirteen leadership projects were proposed and presented.

**Discussion:**

The LI provided present and future leaders throughout Rwanda with exposure to transformative leadership concepts and offered them the opportunity to work together on projects that enhanced their profession and met the needs of underserved communities.

**Constraints and challenges:**

Challenges included limited funding for physiotherapy positions allocated to hospitals in Rwanda, particularly in the rural areas. Participants experienced difficulties in carrying out leadership projects without additional funding to support them.

**Lessons learned:**

While the emphasis on group projects to foster local advocacy and community education is highly recommended, the projects would benefit from a strong long-term mentorship program and further budgeting considerations.

**Conclusion:**

The LI can serve as a model to develop leadership skills and spur professional growth in low-resource settings. Leadership development is necessary to address worldwide inequities in health care. The LI model presents a method to cultivate transformational leadership and work toward improvements in health care and delivery of service.

## Background and Rationale

In low-resource countries, the impact of a few skilled and dedicated leaders is not only essential to ensure the success of short-term projects but also may be the central element in the development of sustainable health programs and systems. In countries with a more recent history of professional-level training, the number of experienced academic and clinical leaders is usually small (1–4). Talented and skilled leaders are promoted rapidly, asked to serve in multiple settings and administrative capacities, and are often over-extended, all factors that can result in burnout or departure from the health-care workforce (5). There is a need to develop leadership skills for those professionals already in leadership positions, and the next generation of leaders.

One of the primary methods of expanding impact is to disperse and disseminate new practices, including the development of leadership skills for both urban- and rural-based professionals. The geographic disparities of professionals in any low-resource country is high (6), and promoting leadership skills for individuals who are most likely to impact their own communities offers added benefits when seeking to transform professional standards and impact (4). The recommendations from a WHO Global Health Workforce Alliance taskforce on education and training included strategies to move learning into the community, expand teaching capacity, integrate training with service, and maximize impact through regional approaches (7). In the same report, the task force advocated developing leadership skills as a component of building health workforce capacity.

Ousman et al. (8) and Ferguson et al. (9) provided successful examples of leadership initiatives with benefits for the medical community. A longitudinal project designed to promote leadership development for nurses by the International Council for Nurses’ Leadership for Change program has been implemented in more than 60 countries over the past two decades. In a review paper, Ferguson et al. (9) described how leadership initiatives resulted in new quality improvement systems, and training programs. They attributed new models of community partnerships along with professional association initiatives to improvements in health-care systems and policy (9). Ferguson et al. (9) also stated the belief that graduates of leadership training were less likely to emigrate, addressing the costly problem of “brain drain” that impacts the entire workforce (5, 7).

Ousman et al. (8) designed an interprofessional fellowship program with the goal of mentoring and training emerging leaders to improve HIV prevention and care in African countries. The initiative funded by the President’s Emergency Program for AIDs Relief provided intensive mentorship and leadership training for physicians, nurses, and public health professionals (8). In addition to fostering partnerships between academic and government entities, a characteristic of this program was encouraging fellows to develop solutions to problems for workplace or community needs to strengthen systems. Ousman et al. (8) summed up these concepts by stating: “Leadership programs in Africa can have a profound effect on expanding a workforce of emerging transformational leaders capable of health systems reform.”

These same types of projects are not firmly established in the rehabilitation literature for professional development in low-resource countries. While some programs have attempted to bolster professional associations and supported individuals to attend conferences or other professional activities (10–13), specific leadership training goals and methods have not been documented.

In 2015, Health Volunteers Overseas (HVO) initiated the Advancement of Rwandan Rehabilitation Services Project (ARRSP) funded by the United States Agency for International Development (USAID). HVO has a long history of developing and implementing capacity building projects to improve health-care provision in low resource settings. The ARRSP program goals included (1) provision of continuing professional development (CPD) courses to Rwandan physiotherapists in order to upgrade rehabilitation standards and (2) increase awareness of the profession of physiotherapy among the general public and other health-care professionals in order to increase utilization of rehabilitation services and reach under-served populations. The course materials and evaluation documents (14) are available through the USAID Clearing House. The full project details, results, and impact are discussed by Dunleavy et al. (15). By design, the ARRSP teaching and learning activities incorporated constructivist approaches as an underlying educational framework and the transformational leadership paradigm as an approach to providing professional development in the areas of leadership and advocacy to physiotherapists from both rural and urban settings in Rwanda. The purpose of this paper is to present an overview of the activities and outcomes of our leadership development module, The Leadership Institute (LI). We believe this model of leadership development may provide guidance for similar trainings in other low-resource countries and that our reflections will offer the educational community an opportunity to build on our model and construct a research agenda around leadership development.

## Pedagogical Framework

Constructivist approaches allow learners to shape their learning based on their own experience, background, beliefs, and prior learning (16, 17). One of the major foundations of this approach is using activities to maximize the backgrounds and skills of participants. The use of constructivist approaches requires instructors to act as facilitators rather than delivering the material in a more passive manner (18). The learner drives instruction while instructors promote problem solving and provide guidance to achieve the desired objectives (19). In order to provide learning opportunities, questions and activities are designed to encourage students to discuss their ideas and solutions (17).

This broad educational philosophy extends into the use of (1) contextual learning concepts centered around application of learning in the learner’s environment and (2) active learning methods that drive learning. Tessmer and Richey (20) suggest that it is essential to consider the surrounding context in order to maximize the relevance, acquisition, and application of knowledge and skills. Contextual learning is designed with specific elements related to the learner’s environment. Active learning methods promote engagement through methods such as projects, discussion, and interaction. These concepts are considered essential for adult learning (19). Engaging in authentic activities in the actual setting or close to the setting in which the skills and information are to be applied is thought to link new concepts with the learner’s strengths and existing knowledge, and promote transfer of skills into the real world. Since one of the goals of the ARRSP was to promote application of knowledge and skills gained in the content oriented courses, the inclusion of active learning assignments such as education and advocacy within the participant’s community were used to promote sustainability and transfer (14).

The development of leaders and leadership development are essential for the growth and advancement of organizations and professions (21). Leadership is crucial to the improvement of health and health care (22). While leader development activities focus on the individual as a leader, leadership development enhances the leadership capacity of an organization (23). There are many theories and concepts used to examine and define leaders and leadership (22, 23). Burns (24) provided a conceptual approach to leadership defining two types of leaders, transactional and transformational. Transactional leaders emphasize the achievements of tasks and expect their followers to meet preset standards. Transformational leaders “stimulate and inspire followers to both achieve extraordinary outcomes and, in the process, develop their own leadership capacity” (25). Swanson et al. (26) suggested the use of transformational leadership will be an essential strategy to meet a global health agenda including the Sustainable Development Goals.

Transformational leadership development approaches are well suited to the advancement and development of a profession since they focus on how a group can share leadership capacity. Bass and Riggio (27) provided examples of, and a context for, successful leadership training. This training can apply to leadership development at the professional association, academic, community, or individual level. Transformational leadership training and assessment has occurred across many cultures, in many professions (25, 27–29).

As the ARRSP grant activities progressed, expanding the emphasis to promote more extensive change as well as leadership development led to the idea of a LI. In this final course, an essential component to facilitate leadership development was the use of active learning projects driven by course participants. The identification and planning of the projects required thinking about needs from a broad perspective, beyond everyday practice. While active learning projects require real problem solving, learning is the primary goal, and often mistakes or early exploration can lead to vital analysis and reshaping of thought processes. These situations not only require leadership skills; the processes of identifying and meeting community needs create opportunities for discussion of ways to create and support change. Group or team learning is an ideal format to develop leadership skills. Kennedy (30) described a continuum of professional development models with the highest level characterized by a transformative purpose where groups include collaborative problem identification that drives exploring and understanding practice. The culminating course strove to challenge and stimulate established leaders and those with leadership potential, using these interactive approaches with the goal of promoting further development of transformative leadership.

### Learning Environment

Physical therapy is a relatively new profession in Rwanda with the first cohort of diploma-trained physiotherapists graduating in 1999. While all physiotherapists now educated at the Bachelor’s Degree level, at the start of the project in 2015, there were minimal opportunities for CPD in the country. Other medical professionals and the general public had a limited awareness or understanding of the scope of physical therapy, contributing to under-utilization of rehabilitative services for individuals with injuries and disabilities.

Physiotherapists in Rwanda work in health care at every level treating diagnoses across the lifespan. Hospitals employ the largest number of physiotherapists. Other practice settings include rehabilitation facilities, outpatient centers, and sports facilities. Physiotherapists can work independently as first-line practitioners, or in a health-care team.

### ARRSP Courses Prior to the LI

**Figure 1:**
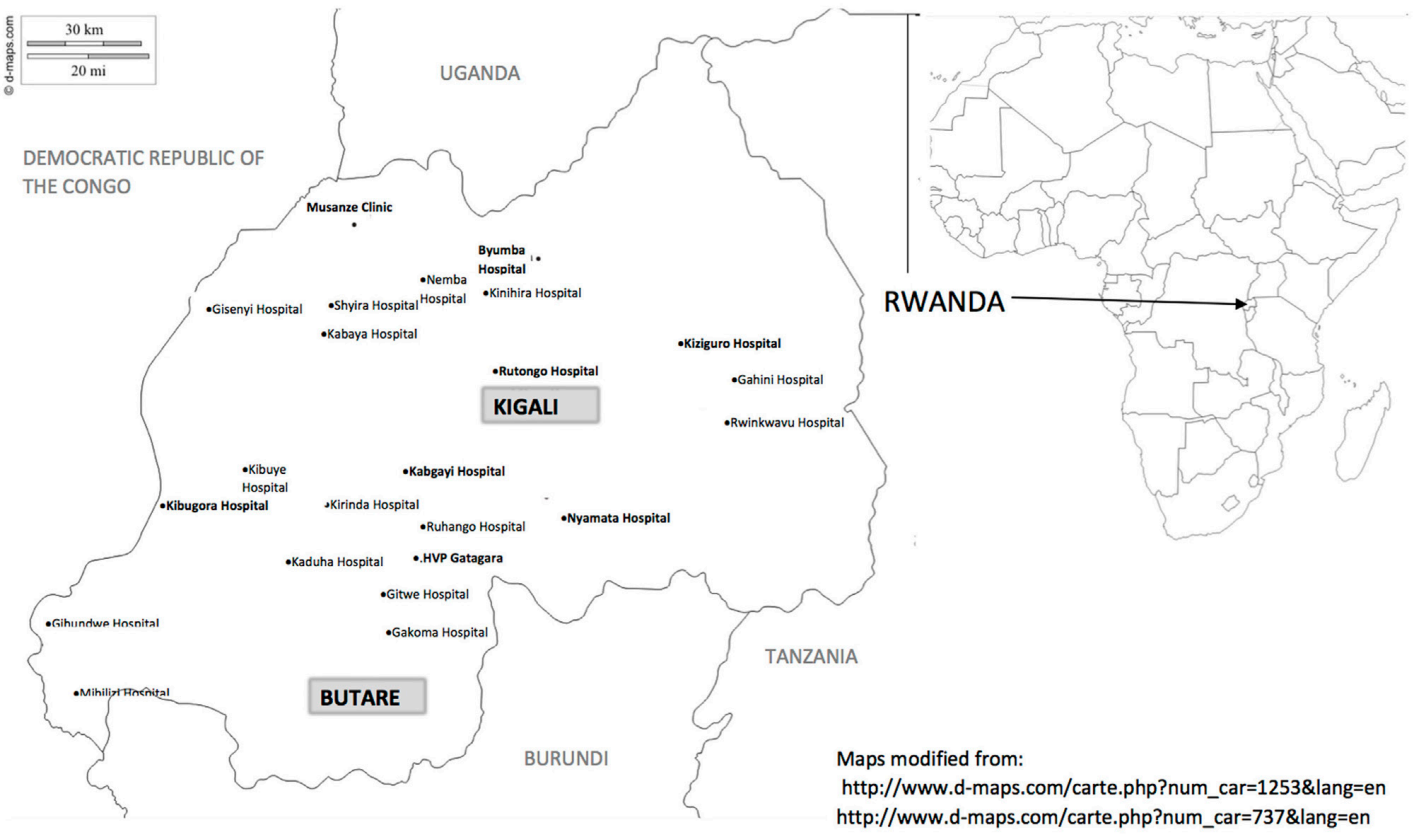
Clinics and hospitals with PTs participating in the Leadership Institute. (Hospitals in Kigali and Butare are not included.)

The first five courses were taught by volunteer content experts from the United States who partnered with Rwandan co-teachers to offer skills-based courses to physiotherapists throughout Rwanda. The courses were offered in the two major academic settings in Rwanda, Kigali, and Butare, facilitating participation from a large geographic area (Figure 1). The didactic and practical courses were supported by clinical site mentoring to encourage application and dissemination of knowledge and skills (14).

### Identification of Need, Development of the LI, and Overall Goals

While advocacy activities were included as part of active learning methods in the skills courses, many course participants expressed a lack of confidence when approaching other health professionals, and some requested guidance in carrying out campaigns to increase the understanding of physiotherapy among the general public. The Rwandan Steering Committee, who contributed to ongoing review of the grant activities, agreed that the focus of the final course would emphasize transformational leadership skills. The emphasis of the LI was to provide development opportunities for current and future leaders with the goal of expanding their impact within their communities.

### LI Learning Objectives

#### Personal Leadership

1. Review and discuss leadership and follower styles.
2. Analyze personal leadership style and reflect on its effectiveness.

#### Professional Leadership

1. Define leadership in the context of physiotherapy.
2. Discuss success in terms of professional goals.
3. Write “SMART” professional goals.
4. Discuss the importance of using evidence in physiotherapy practice.
5. Describe how patient outcomes data can be used to demonstrate individual progress and effectiveness of physiotherapy.
6. Discuss the responsibilities of members of a professional association.

#### Collective Leadership

1. Develop a vision for the physiotherapy profession in Rwanda.
2. Discuss the main purposes of a professional association and its leadership.
3. Identify a challenge related to professional development, awareness of physiotherapy, or an unmet need in the area of physiotherapy.
4. Plan and present a project to meet the identified challenge, including a time frame and budget for implementing the project.

### Pedagogical Format

#### Participants

Seventy-one Rwandan physiotherapists who had attended prior ARRSP CPD courses participated in the LI. Selection criteria included current involvement in a leadership role. Individuals who were heads of physiotherapy departments or private practices, and some more recent graduates who demonstrated leadership at their workplaces received recommendations from course instructors. Individuals from 18 urban and 25 rural settings were invited to participate, with some areas represented by more than one physiotherapist.

#### Organization, Instructional Design, and Implementation

The leadership initiatives were implemented using limited lecture material, interactive discussion, and explicit empowerment of leadership in small group community projects. Support and mentorship was provided for group activities, projects, and presentations. Two cohorts of the LI met on alternating weekends for a total of three sessions (Group A: 36 participants, Group B: 35 participants). All sessions were held at the University of Rwanda College of Medicine and Health Sciences in Kigali.

Content included definitions of leadership with a focus on transformative leadership, and leadership styles consistent with transformative theory. Participants engaged in discussions about qualities that contribute to effective leadership and professionalism. The topics regarding professionalism centered around strengthening the Rwanda physiotherapy association, known as the Association de Kinesitherapie Rwanda (AKR); developing a vision for the future of physiotherapy in Rwanda; developing personal and professional development plans; and the use of evidence-based practice and outcome measures to impact organizational development and clinical practice. Because the physiotherapy community in Rwanda is small, participants were able to use examples of styles and qualities of leadership to which almost everyone in the group could identify. The course instructors worked to facilitate productive analysis and sharing of ideas for improving leadership, asking participants to analyze their own leadership skills and qualities, along with developing plans for improving leadership ability.

Group projects were developed during the course with the intent of implementation after the completion of the LI, to encourage continued growth and development of the physiotherapy profession. Using a constructivist approach, participants identified topics relevant to their own environment and practice, and groups were established based on an area of interest and the needs. The user-defined topics served two goals: the participants developed projects that were important and relevant to their own practice and they were able to develop leadership and advocacy skills related to a real topic.

Feedback from course instructors and other volunteers from HVO was provided throughout the process of leadership project development.

### Formative and Summative Evaluation

Participants completed a test before and after the course to assess knowledge of leadership concepts, attributes of leaders, and their ability to write goals and develop plans. Participants also completed an anonymous survey about the entire ARRSP project that included questions directly related to the goals of the LI.

As part of the project methods, the primary course instructor visited a total of 19 of the 43 participating clinics to mentor professional leadership. Qualitative interviews were completed with 39 physiotherapists. The visits provided information about the context for the projects, as well as opinions about the ARRSP overall. The information was threaded into the LI course activities and provided immediate and realistic feedback.

The primary course instructor conducted follow-up interviews 14 months after completion of the course. A total of 52 therapists were interviewed using semi-structured interviews in several group and individual meetings at clinics, in-person, *via* phone interviews, and electronic interviews using email and mobile apps with chat functions.

### Outcomes

#### Pre–Post Assessment and Participant Opinions of Growth

The proportion of participants who felt that “anyone could be a leader” increased from 60 to 88% at the end of the LI. Some of the most substantial areas of positive change noted were increased knowledge of the profession among other health-care professionals; increased utilization of PT services with a commensurate increase in staffing; and improved documentation standards, with notes for all visits and the use of outcome measures to demonstrate patient improvement.

Many physiotherapists stated the LI inspired and empowered them to be leaders in the field. One of the participants stated: “When I am the leader I have to be the first to influence others in achieving the goal.” Another stated: “Good leaders should give good examples. This sentence helped me a lot,” while a third reflected that: “One needs to be self-motivated, confident, and a role model to motivate others and show the way to the people he is leading.”

The increased sense of individual empowerment resulted in physiotherapists seeking referrals: “After the training, the way of approaching doctors increased … we appreciate it and we are proud of it” and including patients as part of the team: “LI has changed what I do as a physiotherapist by letting me know that my patients should participate in the planned activities by giving their points of view.”

One participant attributed the discussions as being the stimulus for starting a private practice: “Though we did not implement the project, we discussed some important issues, which led me to start a private clinical practice.”

### Project Outcomes

Groups developed a leadership project proposal including objectives, goals, a timeline, and budget. Thirteen project proposals (Table 1) were presented in the final session to individuals from the community, professional association members, academic administrators, faculty, and students from the University of Rwanda.

**Table 1:** Leadership institute projects and results.

Type of project	Project title	Project results
Promoting awareness of physiotherapy services in the community to improve utilization by individuals with disabilities and dysfunction	Increasing awareness of physiotherapy among health-care providers in Kayonza district	One physiotherapist in the Kiziguro district has visited a health center to raise awareness of physiotherapy and reports an increase in referrals to the hospital
	Increasing awareness of physiotherapy services among physicians through interprofessional training in Rwanda district hospitals	Met with physicians and community health workers. Physicians refer children with complicated birth history to physiotherapy before discharge from hospital. Community health workers refer children with suspected developmental delay
	Improvement of community utilization of physiotherapy services through interprofessional education	
	Establishment of online resources for physiotherapists and individuals seeking physiotherapy services	Website is up and running. Working with local web-hosting company. Applied for, but did not receive NGO grant. Secured some funding through a PT student club in the US
Community outreach for prevention and management of injury and disability	Community outreach to children with disabilities and their families	One physiotherapist provided an educational session about developmental delay at her village’s Umuganda. She has seen an increase in pediatric referrals to the district hospital
	Falls prevention programs for older adults	Not completed
	Physiotherapy intervention in fighting non-communicable diseases (NCDs) through sports and physical exercise in Gasabo district	Rwandan Association of Allied Health Professionals hosted an International Conference on NCDs
		Seven Rwandan physiotherapists presented at the conference. The conference chairperson is a physiotherapist
		The AKR has organized healthy walks in Kigali City to raise awareness about NCDs. The walks conclude with stretching, during which physiotherapists discuss the importance of an active lifestyle
Improving standards of physiotherapy practice in Rwanda	Standardized physiotherapy assessment documentation	The PT departments at the University Teaching Hospital of Butare (CHUB) and the University Teaching Hospital of Kigali (CHUK) are working on standardizing documentation, including outcome measures, to help with improved documentation of progress
	Development and implementation of clinical guidelines in physiotherapy practice	The PT staff at CHUB is currently working on developing Rwanda-specific clinical guidelines for rehabilitation after shoulder and hip hemiarthroplasty surgeries
	Recommendations for setting up private practice	In June 2016, the RPTO sponsored a continuing professional development program about entrepreneurship, which was attended by 42 physiotherapists
Prevention and management of workplace injury	Awareness of the role of physiotherapy for prevention and treatment of workplace injuries among public and private policymakers in Rwanda	Not completed
	Postural education and ergonomics assessment	Several PTs mentioned difficulty with obtaining permission to perform these activities as a significant barrier to completion
	Appropriate ergonomics in the working environment: case study—Rwanda Military Hospital, administrative staff	

#### Stakeholder Interviews—Advocacy and Awareness Outcomes

Overall, there was an increase in the number of community outreach activities completed by physiotherapists. The number of facilities involved in community outreach grew to 19, compared with only 7 facilities previously reporting such activities. Examples of outreach activities included talking to community health workers and visiting physicians. One therapist held an educational session at a meeting during her village’s “Umuganda” (monthly community service day). After the completion of the course, participants reported that many physiotherapy departments (74%) had provided educational programs to increase awareness of physiotherapy. Seven departments (37%) provided information to physicians and other hospital staff and reported an increase in referrals and understanding of physiotherapy. Physiotherapists from one hospital met with nurses at health centers to discuss physiotherapy and also reported a subsequent increase in referrals. Several departments and facilities (16%) carried out other activities to raise awareness about physiotherapy.

#### Vision for the Future and Professional Association Involvement

Participants in the LI developed a draft vision statement for physiotherapy in Rwanda: “physiotherapists are recognized throughout Rwanda as health professionals who provide quality care to patients in multiple settings.”

One of the outcomes of the LI was motivation to contribute to the leadership of the physical therapy organization, the AKR. Participants resolved to increase their involvement in meetings and other activities planned by the AKR. The current leaders of the AKR participated in the LI and are applying skills fostered by their involvement in the program. AKR leaders report that there has been increased participation in AKR events. They have organized CPD courses and have petitioned the Rwandan Association of Allied Health Professions to become authorized providers for future continuing education.

## Discussion

The emphasis of the LI was to provide emerging leaders in rural and urban areas with exposure to transformative leadership concepts and opportunities to make a difference within their profession and communities. This final course was sequenced to facilitate sustainability and amplify clinical practice concepts gained in other ARRSP courses. The intention was to promote diffusion of ideas through empowerment and encouragement of advocacy to enhance the growth and visibility of the profession leading to increased accessibility and utilization of services by people with disabilities. The importance of developing a community of practice and a culture of sharing knowledge is a key attribute of professionalism and essential to growth of the profession. The Rwandan physiotherapists participating in the LI embraced this paradigm shift.

In comparison to the leadership projects cited from medicine and nursing (8, 9), this project had fewer participants and was of a shorter duration, making it difficult to equate results. The well-funded medical project related to HIV prevention and care (8) and the longstanding nursing leadership programs (9) concentrated mostly on leadership along with active learning projects. The LI is the first project to our knowledge focusing on rehabilitation leaders. The LI also followed a series of courses targeting practical skills and knowledge, creating a common platform for participants. One strength of this program was the inclusion of both experienced and emerging leaders, reaching 25% of the physiotherapists registered in the country. We believe this reach will contribute to further development of a strong critical mass for future professional initiatives.

The level of community outreach has increased, with physiotherapists taking a lead in internal and interprofessional education. They have advocated for better health outcomes and improvement of services. They have promoted team-based care to promote the quality and quantity of health care. Team-based care can optimize health resources, increase safety, and improve satisfaction among patients and health professionals (9, 31).

While the outcomes of the LI program cannot be separated from foundations laid in earlier ARRSP courses, participants believe that the advocacy and outreach work from the LI has led to an increase in physiotherapy referrals. They generally agreed that this increase in referrals has the potential to further demonstrate the value of physiotherapy, highlight the knowledge of the physiotherapy profession, and provide needed services to a large population of people living with disabilities. Outreach activities have mainly targeted other health-care professionals. Further outreach activities targeting the community at large would increase the awareness of the profession.

One of the characteristics of transformative leadership is the sharing of leadership responsibilities. This was one of the most successful aspects noted during activities. Participants worked together on projects and shared clinical problems and solutions through mobile communication applications such as WhatsApp. Often professionals in the district hospitals are isolated. The LI allowed for networking and discussion for individuals in the rural areas, which was a positive and important step toward upgrading the profession throughout the country. The commitment of the group to expand and support professional association initiatives was also a positive outcome.

It was important to have the input of the Rwandan co-teachers in the design of the activities and the LI projects. The Rwandan course instructors provided valuable cultural insight and anticipated challenges for the projects and plans started during the LI. Many Rwandan physiotherapists have more than one job, working evenings, and weekends. This reduces time and resources available for professional activities. Regardless, the Rwandan course instructors served as role models for juggling professional duty and the need to earn a living. The growth of the co-instructors was notable during the course and when presenting at local and international professional association activities, including the World Confederation of Physical Therapy meeting. They have continued to be role models in community and academic endeavors.

Although not all the active learning projects were implemented, the process of developing group projects with other leaders promoted greater involvement of the participants in education and leadership in Rwandan health care. Even with partial completion, there was a sense of accomplishment, and those who were able to modify the proposals and complete components of the projects have inspired others to increase their professional involvement. These successes need to continue to be shared widely in the PT community.

### Constraints and Challenges

One of the early challenges faced during the LI was the expectation of continued funding for projects and education. The influx of funding after the 1994 genocide may have contributed to this expectation; this challenge is not unusual with funded development activities carried out in low-resource countries. The course instructors discussed the importance of professionalism and volunteering as a component of professional duty, as well as the realistic limitations for financial support. A number of the projects were very ambitious and had to be scaled down. As noted by Ferguson et al. (9), projects developed during a leadership training program are more likely to be successfully implemented if they receive local financial support. Work on the majority of the projects continued despite little or no financial support, demonstrating the willingness of participants to serve as role models for change. The group working to develop a website attempted unsuccessfully to get grant funding, but did secure some funding through a student physical therapy club in the US. Future projects may need to establish expectations up front if there is no funding allocated for micro-grants. Other options are explicit objectives to investigate local resources, budget preparation, management, limits.

Despite the need for physical rehabilitation in Rwanda, there are limited physiotherapy positions allocated to hospitals, particularly in the rural areas. In the district hospitals, every physiotherapy department head reported requesting additional staff at least once in the past year, and being told either to wait, or that a position was not possible financially. Discussions during the LI also highlighted the need to be able to justify new positions. The use of outcomes had been introduced in the skills based courses and was reinforced as a tool to demonstrate the benefit of physiotherapy services. However, the use of outcomes and documentation was a new concept for most of the participants and while there were improvements, this method may take more reinforcement and time to be consistent. Although six clinics reported an increase in staffing at follow-up, nearly all those interviewed felt staffing was insufficient, especially with increased referrals. It is also difficult to determine a causal relationship between staffing and documentation without more extensive study of all factors involved. Nevertheless, continued advocacy for physiotherapy as a necessary service for patient improvement would benefit from data to support patient progress. As discussed by Swanson et al. (26), this is an example of a situation in which transformational, systems level change is needed. The problem was discussed with the leadership of the AKR who agreed this is an issue that should be addressed at a national association level and began discussions of plans to address this through the Ministry of Health. Governmental approval of new positions is unlikely to be changed by the AKR only and will require advocacy from other health professions. Collaborative, interprofessional support will be contingent on physiotherapists continuing to work to increase awareness of their profession.

At the 14-month follow-up, most clinics were performing education about physiotherapy services and benefits within the hospital or clinic setting, but not all were performing outreach in the community. Therapists discussed the need for providing community outreach for both education and provision of direct care to underserved populations. The biggest barriers to community outreach were problems with permission to leave work for these activities and funding for transportation and time. These barriers will need to be addressed both at the institutional level and national association level.

While much progress has been accomplished within the short timeframe, there remain additional challenges to ensure improvement continues. The infrastructure for communication and transportation in Rwanda remains an impediment to having meetings electronically or in person. Although most people have cell phones, they must pay for calls and data on a regular basis. Some, but not all clinicians, have access to the internet and a computer at the workplace. Being able to meet to share successes and struggles will be essential for continued growth of the profession. As with patient care, data about successes and challenges in terms of outreach, staffing, and other management concerns need to be documented and disseminated. The AKR leadership has expressed interest in helping foster communication, a very appropriate and useful role for the organization. A strong professional organization will be paramount to continued progress and success. The use of Umuganda to educate community members about physiotherapy can help to increase access to services. There remains a large underserved population in Rwanda, and using community activities for education has the potential to begin to address this problem.

### Lessons Learned and Suggestions for Future Projects

If a similar teaching and learning model for leadership development were to be implemented in the future, we suggest some important considerations. Introducing the leadership project development earlier would allow more time for mentorship and for the development of in-country mentors. Early mentorship should help participants to narrow the scope of their projects to help ensure completion and to foster implementation with budget limitations. Organizing groups based on geographic proximity can reduce funds needed for meetings, is likely to facilitate more frequent communication, and could be beneficial for long-term support. Regular follow-up by mentors, with a transition to local mentoring by a university or professional organization may also help foster project completion. The projects were an innovative mechanism to promote sustainability and increased emphasis during grant planning might allow a longitudinal approach to amplify the benefits. Building interprofessional advocacy activities into the planning for future grants would be very beneficial, including consultation with other professional organizations. However, the platform of the skills-based courses is essential and the project needs assessment will need to identify the leaders who are most likely to benefit from this type of initiative. Finally, the integration of the emphasis on local advocacy and community education, professional organization development, and the individual development is highly recommended.

## Conclusion

The LI conducted for physiotherapists in Rwanda can serve as a model for other countries with a young and developing profession such as physiotherapy. The series of classes and the development of a project fostered leadership skills empowered therapists to advocate for patients and their profession and spurred professional growth. The LI is an example of applying transformational leadership. The physiotherapists who participated in the classes report increased leadership in their work settings, in the professional association, and in the development and participation in CPD activities.

Leadership development in health-care disciplines is necessary to address worldwide inequities in health care. The LI model is one way to cultivate transformational leadership and work toward improvements in health care and delivery of services. The use of a constructivist approach allows direct and immediate application of skills and concepts and can be an important method to promote continued professional development and advocacy after a program has concluded.

## Ethics Statement

The follow-up study was carried out in accordance with the recommendations of the Misericordia University Institutional Review Board with written informed consent from all subjects. All subjects gave written informed consent in accordance with the Declaration of Helsinki. The protocol was approved by the Institutional Review Board.

## Author Contributions

MP, KD, MM, JC, LK, and AN contributed to the concept, original manuscript, and editing of the paper.

## Conflict of Interest Statement

The authors declare that the research was conducted in the absence of any commercial or financial relationships that could be construed as a potential conflict of interest.
